# An RNA Element at the 5′-End of the Poliovirus Genome Functions as a General Promoter for RNA Synthesis

**DOI:** 10.1371/journal.ppat.1000936

**Published:** 2010-06-03

**Authors:** Dorothee A. Vogt, Raul Andino

**Affiliations:** Department of Microbiology and Immunology, University of California, San Francisco, California, United States of America; Washington University School of Medicine, United States of America

## Abstract

RNA structures present throughout RNA virus genomes serve as scaffolds to organize multiple factors involved in the initiation of RNA synthesis. Several of these RNA elements play multiple roles in the RNA replication pathway. An RNA structure formed around the 5′- end of the poliovirus genomic RNA has been implicated in the initiation of both negative- and positive-strand RNA synthesis. Dissecting the roles of these multifunctional elements is usually hindered by the interdependent nature of the viral replication processes and often pleiotropic effects of mutations. Here, we describe a novel approach to examine RNA elements with multiple roles. Our approach relies on the duplication of the RNA structure so that one copy is dedicated to the initiation of negative-strand RNA synthesis, while the other mediates positive-strand synthesis. This allows us to study the function of the element in promoting positive-strand RNA synthesis, independently of its function in negative-strand initiation. Using this approach, we demonstrate that the entire 5′-end RNA structure that forms on the positive-strand is required for initiation of new positive-strand RNAs. Also required to initiate positive-strand RNA synthesis are the binding sites for the viral polymerase precursor, 3CD, and the host factor, PCBP. Furthermore, we identify specific nucleotide sequences within “*stem a*” that are essential for the initiation of positive-strand RNA synthesis. These findings provide direct evidence for a trans-initiation model, in which binding of proteins to internal sequences of a pre-existing positive-strand RNA affects the synthesis of subsequent copies of that RNA, most likely by organizing replication factors around the initiation site.

## Author Summary

Enteroviruses are a subfamily of small, pathogenic, icosahedral viruses called picornaviruses. Poliovirus, the etiologic agent of paralytic poliomyelitis, is one of the most extensively studied members of this family. Poliovirus RNA replication utilizes a mechanism, common to all positive, single-stranded, lytic RNA viruses, which permits the amplification of a single initial molecule of RNA into thousands of RNA progeny in only a few hours. After entry, the viral genomic RNA is transcribed to generate a complementary RNA (negative-strand), which, in turn, is used as a template to synthesize new strands of genomic RNA (positive-strand). The specificity of the viral RNA template, and the relationship between translation and replication, are controlled by RNA elements present throughout the genome. Individual elements often carry out multiple, interdependent tasks, complicating the dissection of their precise roles in specific steps of replication. We employed a novel approach to overcome this roadblock. Our strategy demonstrated that an RNA element present at the 5′ end of the virus genome is the master regulator of the initiation of RNA synthesis.

## Introduction

The genome of positive-strand RNA viruses has dual functions: as viral mRNA and as template for the synthesis of additional RNA genomes. Replication of the viral RNA occurs by a highly regulated, efficient mechanism, which produces tens of thousands of new RNA copies in only a few hours. Positive-strand RNA viruses follow a common strategy for replication: the viral genome is transcribed into a negative-strand intermediate, which, in turn, acts as a template for new positive-strand synthesis. The same RNA-dependent RNA polymerase synthesizes both RNA strands using viral and host factors. However, replication is a highly asymmetric process, resulting in the synthesis of many more positive- than negative-strands. Accordingly, a regulatory mechanism should exist to control levels of production of either strand, perhaps at the level of initiation.

Several viral RNA structures present within negative- and positive-strand RNA are important for initiation of RNA synthesis. RNA elements that organize RNA replication initiation complexes are believed to form around the 3′-termini of both positive and negative strands, but, surprisingly, they have also been found at the 5′ end of the viral RNA or within the coding region of the virus RNA. Furthermore, recent evidences suggest that interaction of the 5′- and the 3′-ends of the viral genome is necessary to initiate negative-strand RNA synthesis [1], [2], [3], [4], [5]. These 5′-3′ interactions are mediated either by direct RNA-RNA interaction of complementary sequences in the genome [1], [2], [3], [4] or through an RNA-protein-protein-RNA bridge [5]. The global folding of the viral RNA genome could explain how RNA elements, dispersed throughout the genome, could assemble together into a complex that catalyzes initiation of negative-strand RNA synthesis. However, the relationship between structure and function of these complex elements remains poorly understood. A complication in dissecting the precise role of these RNA elements arises from the fact that some of these structures participate in multiple steps of the replication process. Such is the case for a 5′- RNA element in the poliovirus genome that is proposed to contribute to the initiation of both negative- and positive-strand RNA synthesis. The overlapping functions of this element have limited our understanding of its structural and functional features.

Here, we examine the structure and function of the initiation complex of positive-strand RNA synthesis. We chose poliovirus, a member of the family *Picornaviridae*, as a model because both *in vitro* and *in vivo* systems are available to dissect the viral replication cycle [6], [7], [8]. Poliovirus contains a single positive-strand RNA genome of approximately 7500 nucleotides which is covalently linked to a small peptide, VPg, at the 5′-end and contains a poly(A) tail at its 3′-end [9], [10], [11], [12]. The viral RNA consists of an open reading frame flanked by two untranslated regions (UTR), at the 5- and 3′-ends of the genome. The 5′-UTR contains two functional elements important for translation and replication: The internal ribosomal entry site (IRES) region spanning five stem loop structures within the 5′-UTR drives translation of the polyprotein via a cap-independent translation mechanism [13], [14]. The 5′-terminal 94 nucleotides fold into a cloverleaf-like structure, which plays a role in both translation and replication [15], [16], [17]. The cloverleaf structure is a key *cis*-acting element for initiation of negative-strand RNA synthesis [5], [18]. The cloverleaf structure forms a ternary complex with the cellular poly(rC) binding protein (PCBP; also known as hnRNP E or -αCP) [19], [20] and the uncleaved viral precursor of the polymerase, 3CD [15], [16], [17]. This complex can interact with the cellular factor, poly(A) binding protein (PABP), which binds to the poly(A)tail at the 3′-end of the genome. This leads to pseudo-circularization of the viral genome and initiation of negative-strand RNA synthesis [5], [18]. The ternary complex formed on the 5′-cloverleaf structure also functions in translation, thus providing a means to tune the balance between translation and RNA synthesis [21], [22]. The binding of the cellular protein, PCBP, to the cloverleaf RNA enhances viral translation (Gamarnik and Andino, unpublished). In contrast, binding of the viral polymerase precursor, 3CD, to the cloverleaf structure represses translation and promotes negative-strand synthesis of the viral RNA [22], [23].

Initial evidence also implicated the cloverleaf structure as a critical RNA element for positive-strand RNA synthesis. Certain mutations in this element resulted in reduced accumulation of positive-strand RNA without a significant effect on negative-strand levels [16]. This observation raised the question how the cloverleaf structure functions as a promoter for positive-strand RNA synthesis, which is initiated on the 3′-end of the negative-strand. Analyzing the precise role of the cloverleaf element in positive-strand RNA synthesis, however, is difficult because most mutations disrupting the structure and/or functions of the cloverleaf also inhibit negative-strand RNA synthesis.

We thus developed a novel approach to analyze RNA elements that play multiple roles during virus replication. Our approach relies on the duplication of the cloverleaf structure so that one cloverleaf is dedicated to the initiation of negative-strand RNA synthesis while the other can mediate positive-strand synthesis. This allows the study of the function of the cloverleaf RNA on positive-strand RNA synthesis. Our studies demonstrate that the cloverleaf structure formed at the 5′-end of the positive-strand is required for initiation of positive-strand RNA synthesis. Also required to initiate positive-strand RNA synthesis are the binding sites for the viral polymerase precursor, 3CD, and the host factor, PCBP. Furthermore, we identified specific nucleotide sequences within *“stem a”* that are essential for the initiation of positive-strand RNA synthesis

## Results

### Duplication of the 5′ cloverleaf RNA to examine its role in positive-strand RNA synthesis

**Figure 1:**
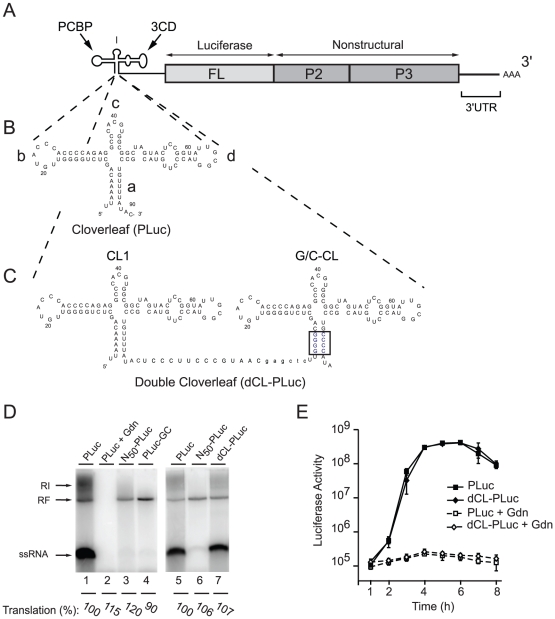
Double cloverleaf replicons. (A) Schematic representation of a poliovirus-luciferase replicon. (B and C) Secondary structure of the PLuc cloverleaf and the two tandem cloverleaves in dCL-PLuc with wild-type sequences in CL1 and four G-C pairs in “*stem a*” of the downstream cloverleaf G/C-CL (highlighted in blue). (D) RNA replication in a cell-free system. RNA transcripts either Pluc (lanes 1, 2+5), N50-Pluc (50 non-polio nucleotides at the 5′-end of the viral RNA) (lanes 3+6), Pluc-GC (lane 4), or dCL-Pluc (lane 7) were used to program HeLa cell S10 extract. After 4 hours of incubation at 30°C, translation levels were measured as luciferase activity (arbitrary units [AU]), and pre-initiation complexes were isolated by centrifugation in the presence of 2 mM guanidinium hydrochloride (Gdn). RNA synthesis was initiated by addition of NTP and monitored by [α^32^P]UTP incorporation for 2 hr. The RNA synthesized was analyzed using native agarose gels. (E) Replication of poliovirus replicon in intact HeLa S3 cells. RNA transcripts (PLuc or dCL-PLuc) were transfected into HeLa S3 cells, and luciferase activity [AU] corresponding to 2.5×10^5^ cells was measured every hour for 8h. The cells were incubated either in the presence (dashed lines) or absence (solid lines) of 2 mM Gdn. Each measurement was carried out in triplicate; standard deviations are indicated by vertical bars.

To examine the role of the cloverleaf structure in positive-strand RNA synthesis we designed an artificial virus RNA genome with two independent RNA replication promoters dedicated to either positive- or negative-strand RNA synthesis (Fig. 1A to 1C). Previous results have shown that only the structure but not the specific sequences of the cloverleaf RNA stems are required for negative-strand synthesis [16]. In contrast, the specific nucleotide sequences of *“stem a”* are critical for efficient positive-strand initiation [24]. Furthermore, additional sequences at the 5′-end of the viral genome also lead to a defect in positive- but not negative-strand RNA synthesis [25]. We exploited these findings to construct a poliovirus luciferase replicon with tandem cloverleaf structures, in which the four A-U pairs in *“stem a”* of the downstream cloverleaf were replaced with G-C pairs (Fig. 1C, G/C-CL). In this construct, the downstream cloverleaf will only be able to participate in the initiation of negative-strand RNA synthesis, leaving the upstream, 5′-most cloverleaf open to the analysis of the elements required for positive-strand synthesis. Using enzymatic structural probing of the tandem cloverleaf structure in dCL-PLuc, we confirmed that the two cloverleaves fold as predicted (Fig. 1C), enabling them to function independently of each other (Fig. S1).

A cell-free system that supports complete poliovirus replication [8], was used to demonstrate that the cloverleaf RNA containing a GC *“stem a”* can only promote negative-strand RNA synthesis, resulting in accumulation of dsRNA replicative form (RF) (Fig. 1D, lane 4, PLuc-GC). The labeled RF RNA observed is composed of the input unlabeled positive-stranded and newly synthesized ^32^P-labeled negative-stranded RNA, thus RF can be taken as a direct measure of negative-strand RNA synthesis. As expected, a replicon containing a single wildtype cloverleaf at the 5′-end of the genome can support both negative- and positive-strand RNA synthesis and produced single stranded RNA (ssRNA) and replicative intermediate (RI), in addition to RF (Fig. 1D, lane 1, PLuc). Addition of guanidine hydrochloride (Gdn), which inhibits viral RNA replication [26], [27], [28], blocked formation of either species (lane 2) demonstrating that the bands observed correspond to *bona fide* poliovirus replication. Strikingly, when a wildtype cloverleaf structure was inserted 5′ from the G/C-CL both negative- and positive-strand RNA synthesis were observed (Fig. 1D, lane 7, double cloverleaf, dCL-PLuc). The level of translation (measured as luciferase activity, and normalized to the level of PLuc) was very similar for PLuc and PLuc-GC indicating that the stability of the virus RNA is not affected for PLuc-GC.

We next monitored replication in intact cells by transfecting PLuc and dCL-PLuc RNA into HeLa cells in the presence and in the absence of Gdn (Fig. 1E). Both constructs displayed identical replication kinetics. Monitoring luciferase activity in the presence of Gdn provides translation level of the input-RNA without replication. dCL-PLuc RNA translated with the same efficiency as PLuc RNA. These results indicate that dCL-PLuc RNA is able to support efficient replication in intact cells as well as in a cell-free system. Given that the downstream cloverleaf is dedicated only to negative-strand synthesis, this double-cloverleaf construct provides a system to study the effect of mutations within the cloverleaf that would affect positive-strand RNA synthesis.

### Positive-strand RNA synthesis requires an intact 5′ end cloverleaf-structure

**Figure 2:**
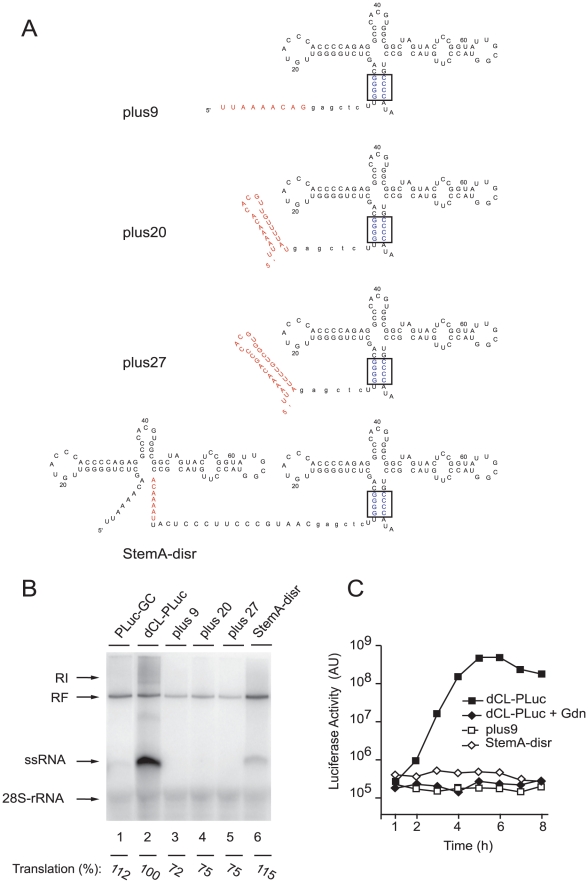
Efficient replication requires a full-length cloverleaf structure at the most 5′-end of the virus genome. (A) Schematic representation of the secondary structure of the 5′- ends of plus9, plus20, plus27, and StemA-disr RNAs. A SacI site (in lower case letters) was introduced as a linker between the partial cloverleaf 5′-ends (in red) and a downstream G/C-CL cloverleaf. (B) RNA replication in a cell-free system. RNA transcripts of either PLuc-GC (lane 1), dCL-PLuc (lane 2), plus9 (lane 3), plus20 (lane 4), plus27 (lane 5), or StemA-disr (lane 6) were used to program a cell extract. (C) Replication of replicons bearing partial cloverleaf structure at the 5′-end. RNA transcripts (dCL-PLuc, plus9 or StemA-disr) were transfected into HeLa S3 cells, and luciferase activity [AU] was measured every hour for 8h. Incubations were carried out in the presence (+Gdn) or absence of 2 mM Gdn. The graphs are representative of three independent experiments.

We next determined the specific 5′-terminal sequences critical for efficient initiation of positive-strand RNA synthesis. First, we determined the minimal 5′ sequences required for positive-strand synthesis by inserting fragments of increasing length corresponding to the wildtype poliovirus 5′-end genomic RNA upstream of the mutant G-C cloverleaf that only drives negative-strand synthesis (Fig. 2A). Given that the downstream G-C cloverleaf (G-C/CL) promotes negative-strand RNA synthesis at wildtype levels (Fig. 1D, lane 4), this experiment addressed the specific requirements for the initiation of positive-strand synthesis. We examined three constructs (Fig. 2A): a construct containing only the poliovirus 5′ most 9 nucleotides plus a linker that facilitated cloning (plus9), a construct in which the wildtype *“stem a”* was inserted (plus20), and a construct carrying *“stem a”* and *“stem c”* in front of the G-C cloverleaf (plus27). In the cell-free replication system these constructs were unable to produce positive-strand RNA (Fig. 2B, lanes 3 to 5). We observed relatively similar levels of negative-strand synthesis (within 2-fold from dCL-pLuc control) and translation (between 72 to 115% of dCL-PLuc translation control) for all partial cloverleaf constructs (Fig. 2B). Furthermore, a construct carrying the first 81 poliovirus wildtype nucleotides, in which the formation of *“stem a”* was disrupted, was also significantly defective in positive-strand RNA synthesis (Fig. 2B, lane 6).

*In vivo* experiments were consistent with these results. Replication of plus9 and StemA-disr replicons were impaired in intact HeLa cells, while the efficiency of translation was comparable to dCL-PLuc in the presence of Gdn (Fig. 2C, dCL-PLuc + Gdn). These findings establish that not only specific 5′-sequences but also the entire structure of the 5′-cloverleaf is required for efficient positive-strand synthesis.

### Features of the cloverleaf structure involved in positive-strand RNA synthesis

**Figure 3:**
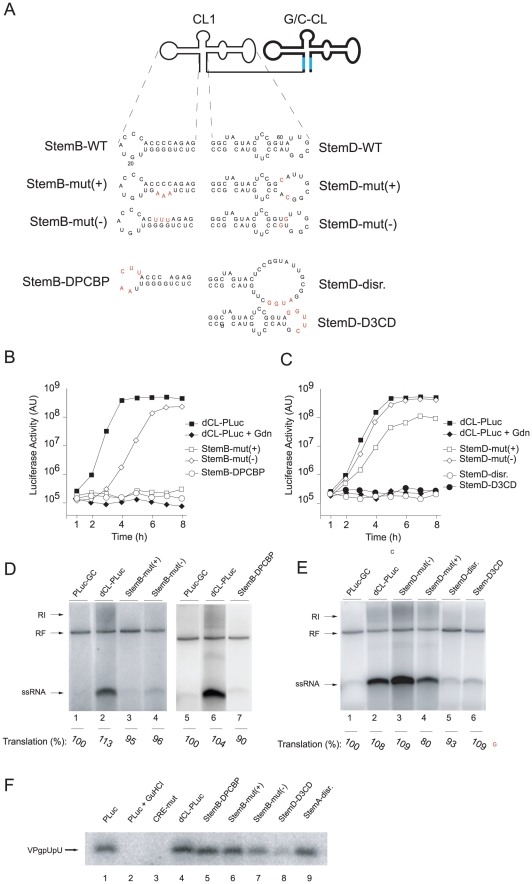
Elements within StemB or StemD required for RNA replication. (A) Representation of the secondary structure of tandem cloverleaf replicons with either StemB or StemD mutations (highlighted in red) in the 5′-end cloverleaf. (B and C) Luciferase expression in replicon RNA-transfected HeLa S3 cells. RNA transcripts containing mutations either within StemB or StemD were transfected into HeLa S3 cells, and luciferase activity [AU] was determined every hour for 8h. Control experiment included the addition of 2 mM Gdn. The graphs are representative of three independent experiments. (D and E) RNA replication in a cell-free system. Replicon RNA transcripts with mutations in either StemB or StemD were used to program a cell extract. (F) VPg-uridylylation in a cell-free replication system. RNA transcripts corresponding to tandem cloverleaf replicons containing mutations in either StemB or StemD were employed to program cell-free replications systems. VPg-pU(pU) formation was monitored by incubating the extracts with [α^32^P]UTP for 1 hour. The radiolabeled RNA was immuno-precipitated using anti-VPg antibodies, separated on a Tris-Tricine SDS-Page gel and visualized by using autoradiography.

Bioinformatic analysis predicts the formation of a cloverleaf secondary structure either at the 5′-end of the positive-strand or at the complementary 3′-end of the negative-strand [16]. Since positive-strand RNA synthesis initiates at the 3′-end of the negative-strand template it has been proposed that the complementary cloverleaf structure in the negative-strand functions as a promoter of the initiation reaction [29], [30]. To examine this possibility, we used a particular property of G-U base pairs to selectively disrupt the structure in the positive- or the negative-strands. G-U pairs can replace A-U pairs in one strand while on the complementary strand the A-C base pairs cannot form, compromising the structure of the stem. We used this approach to incorporate G-U/A-C regions to selectively disrupt either the *“stem b”* or *“stem d”* regions of the 5′-cloverleaf of the positive- or negative strands (Fig. 3A). These were then tested in the context of the tandem cloverleaf replicons, to evaluate the positive-strand synthesis requirements for cloverleaf structure in either (+) or (−) strand.

First, we applied the asymmetric mutational analysis to *“stem b”* by mutating three consecutive base-pairs. In StemB-mut(+), the sequence GGG was replaced by AAA. This mutation should disrupt the stem in the positive-strand but should maintain the structure in the negative-strand. The second construct, StemB-mut(−) (CCC to UUU) disrupted the duplex structure in the negative-strand without modifying the structure in the positive-strand. While StemB-mut(+) did not produce any detectable positive-strand RNA, StemB-mut(−) was able to produce small, but detectable amounts of ssRNA. Strikingly, in intact cells, StemB-mut(+) was not able to replicate, whereas, StemB-mut(−), after an initial delay, replicated with almost wildtype kinetics, reaching similar maximum luciferase expression (Fig. 3B). The slight delay in StemB-mut(−) replication and the reduced level of positive-strand synthesis in the cell free system (Fig. 3D, lane 4) likely results from a weaker G-U base-pairing compared with the wildtype G-C base-pairs, implying that the function performed by the positive strand is sensitive to the stability of the stem. These results indicate that positive-strand RNA synthesis requires an intact *“stem b”* in the cloverleaf structure of the positive-strand RNA.

In *“stem d”* we first introduced mutations that disrupted base-pairing interactions (Fig. 3A, StemD-disr.). This mutant was unable to replicate *in vivo* or *in vitro* (Fig. 3C and 3E, lane 5). We then replaced two A-U pairs with A-C pairs, resulting in disruption of the *“stem d”* only on the positive-strand (StemD-mut(+)), or two G-U pairs (StemD-mut(−)), which should maintain the duplex structure on the positive-strand but should alter the structure on the negative-strand. In the cell-free replication system, StemD-mut(+) showed a reduced level of positive-strands as compared to dCL-PLuc (Fig. 3E, lane 2 and 4). In contrast, StemD-mut(−) was able to efficiently synthesize positive-strand RNA, resulting in even two-fold increase in positive-strand accumulation (Fig. 3E, lane 2 and 3). Our analysis in intact cells was consistent with the *in vitro* results. StemD-mut(−) replicated with wildtype kinetics and maximum luciferase expression, whereas, StemD-mut(+) showed a 10-fold decrease in replication (Fig. 3C).

Importantly, the defects in positive-strand synthesis were not due to decreased RNA stability because we observed no decrease in translation in any of the systems. The level of luciferase activity as an indirect measure for translation was approximately the same for all the mutants in comparison to wild-type in the cell-free replication system (see for example Fig. 1D, 2B, 3D and E). In addition, after transfection into cells, we monitored luciferase activity in the presence of Gdn for all mutants, which provided translation levels produced by the input RNA. Translation level for each mutant was within 10% of wildtype (data not shown). Therefore, we exclude RNA stability as an explanation for the decrease in positive-strand RNA accumulation. These results, as well as those obtained for *“stem b”*, indicate that compromising the cloverleaf structure on the positive-strand RNA leads to a defect in initiation of positive-strand synthesis, as originally suggested [16].

### Binding-sites for PCBP and 3CD in the cloverleaf are also required for positive-strand RNA synthesis

The cloverleaf structure interacts with the host-cell factor, PCBP [19], [20], and the viral polymerase precursor, 3CD [15], [16], [17], to form a ternary complex that participates in initiation of negative-strand synthesis [5], [18]. Having defined the structural requirements of the cloverleaf RNA for positive-strand synthesis, we then examined whether binding of PCBP or 3CD is required for positive-strand RNA synthesis. A poly(C) stretch within the “*stem b*” of the cloverleaf has been identified as the binding-site for PCBP [19], [20]. We introduced a mutation that completely disrupts PCBP binding in the 5′-cloverleaf [19]. This mutant, StemB-ΔPCBP, showed a severe defect in positive-strand RNA synthesis in the cell-free replication system (Fig. 3D, lane 7) and in intact HeLa cells (Fig. 3B). We also engineered a deletion within the *“stem d”* region of the 5′-cloverleaf to disrupt 3CD binding [19]. This mutant, StemD-Δ3CD, was also severely impaired in its ability to replicate in the cell-free system (Fig. 3E, lane 6) and in intact cells (Fig. 3C). This result demonstrates that binding of PCBP and 3CD to the terminal cloverleaf structure is required for initiation of positive-strand synthesis.

The uridylated peptide VPg-pUpU functions as a primer for both negative- and positive-strand synthesis [31], [32], [33], [34]. An additional *cis*-acting replication element (CRE) within the 2C-coding region of the poliovirus genome functions as a template for the covalent linkage of two UMP nucleotides to the viral peptide, VPg, resulting in VPg-pUpU [35], [36], [37], [38]. Since evidence has been obtained for a role of the cloverleaf RNA in VPg-uridylylation [39], and given the severe defect in positive-strand synthesis exhibited by some of the mutants examined here, we analyzed whether the primary defect in our mutants impaired CRE(2C)-mediated VPg-uridylylation. HeLa S10 extract was programmed with replicon RNA and VPg-pUpU formation was analyzed by polyacrylamide gel electrophoresis. We examined the total amount of VPg uridylylation by specific immunoprecipitation using polyclonal anti-VPg-antibodies. Extracts programmed with PLuc RNA accumulated VPg-pUpU over the 2 hr incubation time, but Gdn prevented VPg uridylylation (Fig. 3F lane 1 and 2). In addition, a CRE-mutant virus RNA, which carries a mutation within the CRE(2C) region (A_5_ to C mutation) [31], was unable to catalyze VPg-pUpU formation (Fig. 3F, lane 3). These control experiments demonstrated that authentic VPg-pUpU was synthesized under our experimental conditions. As expected, dCL-PLuc also synthesized VPg-pUpU at wildtype levels (Fig. 3F, lane 4). VPg-pUpU formation was also observed in double cloverleaf replicons carrying mutations in the 5′ cloverleaf that either disrupted the *“stem b”* duplex structure on the positive- or the negative-strand (StemB-mut(+) and StemB-mut(−)), the *“stem a”* structure (StemA-disr) or the binding-site for PCBP (StemB-ΔPCBP) (Fig. 3F, lane 5–9). We observed a slight decrease of VPg-pUpU formation in the case of StemD-Δ3CD (Fig. 3F, lane 8). However, this reduction was not consistently observed from experiment to experiment (see Fig. S2). We concluded that the striking defect in positive-strand synthesis observed in cloverleaf mutants is not due to a defect in VPg-pUpU formation.

### Specific sequence within *“stem a”* are essential for positive-strand synthesis

**Figure 4:**
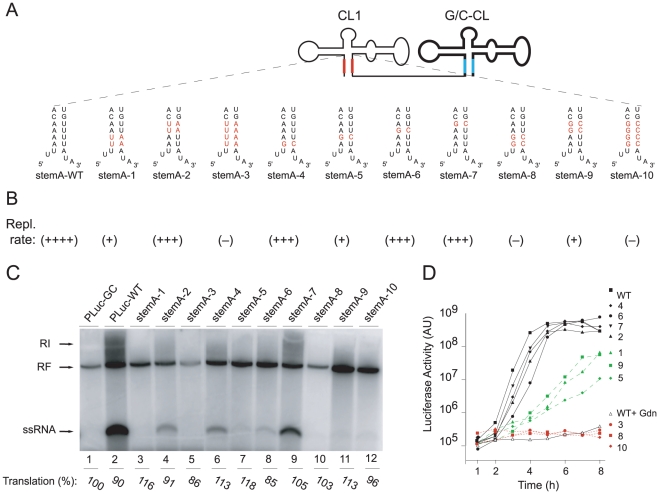
Replication of Double Cloverleaf replicons bearing mutations within StemA. (A) Schematic representation of the secondary structure of Tandem cloverleaf structures replicons with either StemA mutations (highlighted in red) in the 5′-end cloverleaf. (B) Summary of replication rates of StemA mutants in a cell-free system and in HeLa S3 cells. (C) RNA replication in a cell-free system. RNA transcripts containing StemA mutations were used to program a cell extract. RNA products were analyzed on native agarose gels and detected by autoradiography. (D) Replication of virus RNA containing mutations within the StemA. Luciferase activity [AU] corresponding to 2.5×10^5^ transfected HeLa-cells was measured every hour for 8h. The graphs are representative of three independent experiments.

It was previously shown that *“stem a”* of the cloverleaf is needed for positive-strand synthesis [24]. We next defined the specific nucleotide sequence requirements at the *“stem a”* structure for positive-strand synthesis. We engineered a series of *“stem a”* mutations within the 5′- cloverleaf of the double cloverleaf construct. Since disrupting the cloverleaf structure itself leads to a decrease in the level of positive-strand synthesis, sequence alterations were introduced together with compensatory mutations in the opposite side of the “*stem a*” to preserve the structure. The two 5′-terminal uridines were not mutated as they served as template for the 3′-terminal two A's on the negative-strand, which are believed to function as the binding site of the primer VPgpUpU. Instead we mutated a series of four A-U base-pairs (Fig. 4A). All mutated tandem cloverleaf RNAs were tested for positive-strand synthesis in the cell-free replication system and transfected into HeLa cells to examine their replication phenotype *in vivo*. We observed three types of replication phenotypes (Fig. 4B). One group of mutants had a mild effect on the replication rate ((+++) in Fig. 4B). These include mutants where the upper two A-U pairs were changed to U-A pairs, or either the first (starting from the lowest pair), third or fourth A-U pair replaced with G-C pairs (StemA-mut2, -mut4, -mut6, -mut7, respectively). In the cell free system, these mutants synthesized positive-strand RNA, although at a markedly reduced level (Fig. 4C, compare wildtype lane 2 with mutants in lane 4, 6, 8, 9). In intact cells, these mutants showed almost wildtype replication kinetics (Fig. 4D, black lines). The second group of mutants had a severe defect in positive-strand synthesis ((+) in Fig. 4B). These mutants include those where the lower two A-U pairs were replaced by U-A pairs, the second A-U pair was replaced by G-C, or the upper two A-U pairs were replaced with G-C pairs (StemA-mut1, StemA-mut5 and StemA-mut9, respectively). This group of mutants was unable to produce positive-strand RNA in the cell-free system (Fig. 4C, lanes 3, 7, 11), and showed significantly decreased, but still detectable, replication in intact cells (Fig. 4D, green lines). Lastly, some mutants had a dramatic loss-of-function phenotype *in vitro* and *in vivo* ((−) in Fig. 4B). These included the mutants with the most severe alterations in base-pairing. In StemA-mut3 all four A-U pairs were swapped into U-A pairs, in StemA-mut8 the lower two A-U pairs were replaced by G-C pairs, and in StemA-mut10 all four A-U pairs were replaced by G-C pairs. None of these mutants were able to synthesize detectable levels of positive-strands in cell extract (Fig. 4C, lanes 5, 10, 12) or in intact cells (Fig. 4D, red lines). These results establish that changes either in the upper and lower part of the *“stem a”* results in a defect in positive-strand synthesis. However, it seems that the bottom of the stem is more susceptible to sequence changes than the upper portion.

### Reversion of *“stem a”* mutations reveals key role of nucleotide A4 of poliovirus genome

**Figure 5:**
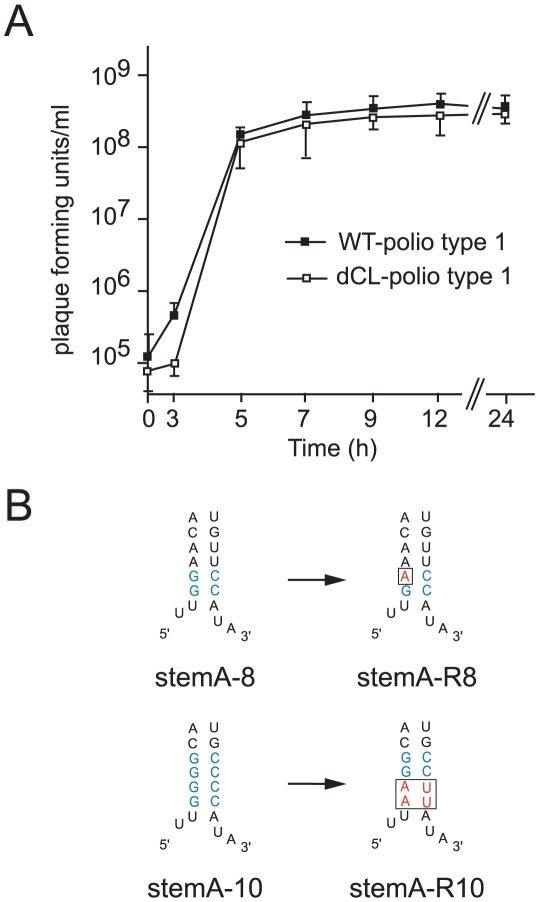
Analyzing the evolution of poliovirus carrying lesions within Stem A. (A) One-step growth curve of poliovirus carrying either one cloverleaf (WT-polio type1) or tandem cloverleaf structures (dCL-polio type 1). HeLa S3 cells were infected at an MOI of 10 with either WT-polio type 1 or dCL-polio type 1 viruses. At indicated time-points viruses were harvested and their titers were determined according to standard plaque assays. The graphs are the mean of triplicate samples. Standard deviations are indicated by error bars. (B) Schematic representation of the cloverleaf StemA-8 and StemA-10 mutations (mutations highlighted in blue) and the changes in sequence observed in revertants, stemA-R8 or stemA-R10 (highlighted in red), with increased replication capacity. Multiple plaques from viruses with higher replication capacity were sequenced and they all contained the mutations highlighted in the figure.

To further define the function of *“stem a”* in positive-strand RNA synthesis, we engineered the full-length poliovirus genome to carry two tandem cloverleaf structures (dCL-polio 1). A one-step growth curve demonstrated that dCL-polio 1 replicates with almost identical kinetics to wildtype poliovirus (Fig. 5A). Furthermore, the plaque-phenotype of dCL-polio 1 was identical to wildtype (not shown). We confirmed by RT-PCR and sequencing that the structure of the double cloverleaf of dCL-polio 1 was maintained during the entire course of the infection (data not shown). Thus, the full-length virus carrying the double cloverleaf replicates with wildtype characteristics and this construct provides a system to study the evolution of a virus carrying a mutated *“stem a”* during normal poliovirus replication in tissue culture.

We examined two different mutations introduced in *“stem a”* of the 5′ terminal cloverleaf, StemA-mut8, and StemA-mut10. These mutations lead to a complete disruption of positive-strand synthesis (Fig. 4). However, poliovirus RNAs carrying these mutations were pseudo-infectious. Following high efficiency transfection with StemA-mut8 and StemA-mut10 viral RNA, we observed viral plaques, albeit at much lower efficiencies than for wildtype RNA. StemA-mut8, in which the lower two A-U pairs of *“stem a”* were replaced by G-C pairs, produced small plaques. StemA-mut10, in which all four A-U base pairs of *“stem a”* were replaced with G-C pairs, displayed a minute-plaque phenotype. We hypothesized that nucleotide changes accumulated after transfection of the mutants increased virus fitness allowing plaque formation. In order to identify the changes that allow these mutants to replicate, the complete genomes of several plaque-purified StemA-mut8-viruses and StemA-mut10-viruses were isolated and analyzed by sequencing. In StemA-mut8, a single point mutation was identified in all independently isolated revertants: a G to A transition in the second G-C base pair (Fig. 5B). Intriguingly, this sequence alteration, which allows the mutant to replicate, results in one mismatch within the *“stem a”* duplex structure (Fig. 5B), suggesting that the sequence is critical for initiation. Strikingly, the StemA-mut10 gain-of-function required a massive four point mutation, which was observed in all isolated viruses, whereby the lower two G-C base-pairs reverted back to wild-type A-U pairs (Fig. 5B). These results confirmed the significance of the specific sequences of *“stem a”* for positive-strand RNA synthesis. Furthermore, they imply a critical role for nucleotide A4 of the poliovirus genome in the initiation of positive-strand RNA synthesis.

## Discussion

Viral RNA genomes often contain RNA elements that fulfill multiple regulatory roles. This is due in part to the compactness of the genome and the need to coordinate various functions of the viral RNA as both genome and mRNA. Dissecting the role of these multifunctional elements is usually hindered by the interdependent nature of the viral replication processes often associated with pleiotropic effects of mutations. This has been the case for the functional analysis of the enterovirus 5′-cloverleaf structure as well as for many other RNA elements in RNA viruses. Here we developed a broadly applicable experimental strategy to circumvent these problems and applied it to examining the role of the 5′-cloverleaf of poliovirus in positive-strand RNA synthesis. Our approach involved engineering of poliovirus replicons carrying tandem duplicated cloverleaf structures at their 5′-ends in which the downstream cloverleaf is defective for positive-strand synthesis but can initiate negative-strand synthesis. This leaves the 5′ most cloverleaf as the only RNA element that can participate in initiation of positive-strand RNA synthesis. This set-up enabled us to directly examine the effect of mutations in the 5′-end structure on positive-strand synthesis. A similar approach could be employed to examine the functional role of RNA elements of other virus families provided the structure can be dissected using mutations that disrupt specific functions.

Interestingly, bovine enteroviruses have two cloverleaf-like structures at the 5′-end of their genome [40]. Deletion of either one of them results in non-viable viruses. However, after exchange of the region spanning both cloverleaves with the coxsackievirus B3 (CBV3) cloverleaf a viable chimera was generated [40]. Thus, the two cloverleaf structures in bovine enteroviruses display the same roles as the single cloverleaf in CBV3, suggesting that one cloverleaf might function as a promoter for negative-strand and the other as a promoter for positive-strand RNA synthesis.

### Functional dissection of cloverleaf regions implicated in positive-strand synthesis

Poliovirus genomes carrying duplicated cloverleaves are fully capable of initiating negative-strand RNA synthesis, and the stability of the positive-strand RNA is maintained by the downstream, intact cloverleaf even if the first structure is disrupted (Fig. 3 and 4). However, our study shows that mutations that disrupt either the structure of, or the binding of factors to the cloverleaf RNA result in reduced positive-strand accumulation (Fig. 4), as previously proposed [16].

Indeed, asymmetric mutations that disrupt *“stem b”* and *“stem d”* in either the positive-or the negative-strand RNA demonstrate that the cloverleaf structure formed in the positive-strand is directly involved in initiation of positive-strand synthesis (Fig. 3B–E). It has been proposed that the stability of viral RNAs carrying mutations within the cloverleaf structure could affect the positive-to-negative RNA ratio. If this were the case, we would expect a decrease in translation of the RNA. However, all mutants reached levels of translation similar to the wild-type RNA (data not shown). Therefore, we exclude RNA stability as an explanation for the decrease in positive-strand synthesis. Our results also show that the interaction of PCBP and 3CD with the cloverleaf is required for efficient positive-strand synthesis. We thus conclude that the same ternary complex used for negative-strand synthesis also plays a critical role in positive-strand synthesis.

Our analysis shows that the duplex structure and sequence of *“stem a”* in the cloverleaf is required for positive-strand synthesis. Disrupting *“stem a”* of the cloverleaf structure results in a virus lethal phenotype (pDNC-91) [16]. More recent studies demonstrated that *“stem a”* is also necessary for negative-strand synthesis [24]. Here, we extended those observations by showing that disruption of four base-pairs in *“stem a”* leads to a severe defect in positive-strand synthesis in the cell free system and completely abrogates replication in intact cells. Furthermore, the specific sequences of *“stem a”* are required for positive-strand synthesis (Fig. 4C), but not for negative-strand initiation. Examining the evolution of mutations that severely affect positive-strand RNA further defined the sequence requirements of *“stem a”*. One single change (A), at the second base-pair of “*stem a*” suffices to greatly increase the fitness of a mutant with a severe replication defect (Fig. 4, stemA-8). This result was consistent with the observation that mutating the second A-U base-pair to G-C resulted in dramatic defect in replication (compare stemA-4 and stemA-5). Interestingly, it appears that in this context a one base-pair disruption can be tolerated. Our results, however, cannot distinguish whether the sequence specificity of *“stem a”* is required in the positive-stranded cloverleaf or in the 3′-end of the negative-strand template. Given that the cloverleaf structure does not require specific sequences at *“stem a”* to facilitate initiation of the negative-strand synthesis, and assuming that the requirements for initiation complex formation are similar for negative-strand and positive-strand RNA synthesis, we propose that the specific sequence at “*stem a*” is required either during unwinding of positive- and negative-strand RNA or at the 3′-end of the negative-strand template initiation site.

### An integrated model for enterovirus replication

**Figure 6:**
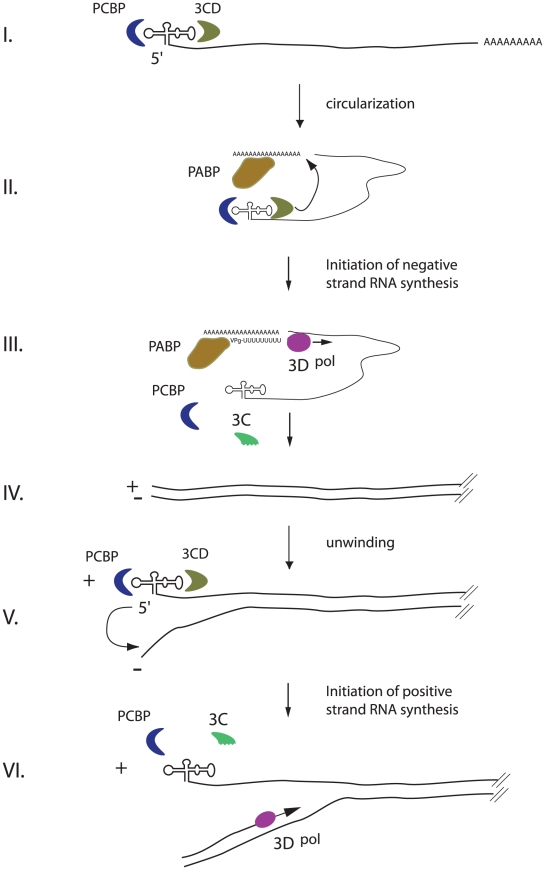
An integrated model for enterovirus replication. Negative-strand synthesis is initiated by circularization of the positive-strand genome via a protein-protein bridge through the interaction of the ternary complex at the 5′-end (3CD and PCBP bound to the cloverleaf structure) and PABP bound to the 3′-poly(A)tail (I. + II.). CRE-mediated VPg-pUpU acts as primer of the reaction and the polymerase 3D synthesizes the new negative-strand (III.), resulting in a double-stranded intermediate (RF) (IV.). The positive-negative duplex RNA intermediate unwinds, so that the cloverleaf structure at the 5′-end of the positive-strand can form. 3CD and PCBP bind to the cloverleaf to form a ternary complex, which, in turn, will initiate positive-strand synthesis on the 3′-end of the negative-strand (V.). The primer, VPg-pUpU, is recruited and binds to the 3′-terminal AA of the negative strand, and the new positive-strand is synthesized by the polymerase, 3D (VI.).

A surprising corollary of our experiments is that the same RNA element at the 5′-end of the positive-strand functions as a promoter for both negative-strand and positive-strand synthesis, which is initiated at the 3′-end of the complementary strand. We propose a model that integrates all available data and explains how the same ternary complex formed around the cloverleaf structure can carry out a bifunctional role in two differently regulated steps during virus replication (Fig. 6). Negative-strand RNA synthesis is initiated by the circularization of the positive-strand genome via protein-protein bridge formed by the interaction of 3CD and PCPB, bound to the 5′-cloverleaf structure, with PABP associated with the 3′ poly-A tail. The cloverleaf recruits the polymerase as an uncleaved precursor, 3CD, via an RNA-protein interaction mediated by a domain within 3C. Active RdRp 3D is either locally produced by autoprocessing of 3CD or recruited by interactions with the 3D domain of 3CD. The peptide-nucleotide primer, VPg-pUpU, is produced with the assistance of CRE, which acts as template. VPg-pUpU primes the reaction and elongation proceeds resulting in a double-stranded intermediate (RF). In order to allow positive-strand synthesis, the cloverleaf RNA must fold at the 5′-end of the positive-strand RNA, thus the positive-negative duplex RNA intermediate must unwind. We hypothesize that a helicase should catalyze this step. It has been suggested that poliovirus protein 2C could carry out this role [41], [42]. 2C and its precursor 2BC interact with the 3′-end of the negative-strand [29], [43], and bioinformatic analyses demonstrated that the 2C nucleoside triphosphate binding domain belongs to the DEAD-box family of helicases. However, 2C has not been directly shown to have a helicase activity and the precise role of 2C during RNA synthesis is still ill-defined. On the other hand, it is also possible that the perfect double stranded RNA at the ‘left end” of the RF can spontaneously unwind to allow formation of the cloverleaf. Further investigation is necessary to define this critical step of enterovirus replication.

Unwinding of the strands is a critical prerequisite for the formation of the cloverleaf structure on the 5′-end of the positive-strand. Following unwinding, PCBP and 3CD could then bind to the cloverleaf structure of the positive-strand. Our data suggest that this step is key for positive-strand synthesis. This should stabilize the cloverleaf structure as well as to keep the 3′-end of the negative strand single-stranded and available for the primer. The primer, VPg-pUpU, is recruited and binds to the 3′-terminal AA of the negative-strand. The simplest model for the initiation of positive-strand synthesis, is that the cloverleaf ternary complex is the organizing element that facilitates the delivery, *in trans*, of the RdRp polymerase, 3D^pol^, from a preexisting positive-stranded cloverleaf to the initiation site, i.e. the 3′-end of the negative-strand template. Once positive-strand synthesis is initiated and the polymerase moves along the negative-strand, this nascent positive-strand will form double-stranded RNA until it is unwound. Unwinding the end is required to form the new cloverleaf ternary complex to initiate a new round of positive-strand synthesis. This mechanism may also determine the observed asymmetry of replication in which more positive- than negative-strands are made. One possible explanation is that the local concentration of cloverleaf at the positive-strand RNA initiation site results in a more efficient reaction compared to that of 7500 nucleotide downstream where negative-strand RNA initiates.

Why does a single RNA element function to initiate both negative and positive strand synthesis? Perhaps the underlining organizing and mechanistic principles are conserved for the initiation of negative and positive strand synthesis and thus the *cis*-acting elements involved in the processes, like the 5′-cloverleaf, are also conserved. However, because in infected cells positive strand RNA accumulates at much higher levels than negative strand, additional regulatory elements must exist to control the efficiency of each process. Accordingly, the cloverleaf RNA would act as a general promoter and additional elements would function as enhancers or regulators of the process. From an evolutionary point of view, it is possible to imagine that a single promoter element first evolved to facilitate initiation of RNA synthesis and then the process was optimized by the addition of regulatory elements. A mechanistic advantage of the proposed model is that by assembling the initiation complex in the positive strand, rather than in the 3′-end of the negative strand, RNA synthesis can initiate and proceed on a free negative strand template without interruptions. Considering these potential advantages of a *trans*-initiation model, it is possible that similar mechanisms may be at work on other positive-stranded RNA viruses. Further experiments in this area are warranted to establish the general characteristics of positive RNA virus replication.

## Materials and Methods

### Cells & viruses

HeLa S3 cells (ATCC CCL 2.2) were grown either (i) in tissue culture flasks in Dulbecco's modified Eagle medium-nutrient mixture/F-12 (Ham) (1∶1), supplemented with 2mM L-glutamine, 100 U of penicillin and streptomycin per ml, and 10% newborn calf serum or (ii) in suspension in suspension minimal essential medium (Joklik modified) supplemented with 2 mM L-glutamine, 100U of penicillin and streptomycin per ml, and 10% newborn calf serum. For virus production of rib(+)Xpa and double-Wt, *in vitro* RNA transcripts were electroporated into HeLa S3 cells under the same conditions as described in the section “RNA transfection for luciferase timecourses”. After electroporation 5 volumes of medium was added. Cells were then incubated at 37°C and 5% CO_2_ over night. After three freeze/thaw cycles viruses were further purified through centrifugation and stored at −80°C (P0 virus). The titers of the virus were determined according to standard plaque assays [44].

### Plasmid design and RNA transcripts

PLuc-RNA is transcribed from prib(+)Luc-Wt, the luciferase-expressing, poliovirus-derived replicon; and WT-polio type 1-RNA is transcribed from prib(+)Xpa, containing the cDNA of the Mahoney strain of poliovirus, as previously described [25]. prib(+)Luc-Wt was used to introduce four G-C pairs in “*stem a*” of the cloverleaf (nucleotides A_3_-A_6_ of the poliovirus sequence were replaced by GGCC and nucleotides U_91_-U_94_ were replaced by GGCC) which resulted in pPLuc-GC. The complementary sequence in the hammerhead ribozyme was also altered to ensure efficient cleavage. A SacI-site was then introduced in front of the poliovirus sequence which resulted in pGC-SacI. The poliovirus Wt-sequence U_1_-C_112_ was then cloned into pGC-SacI, in front of the GC-pair cloverleaf, using the SacI-site, resulting in pdCL-PLuc. pGC-SacI was used as the parental construct for pPlus9, pPlus20, and pPlus27. The following sequences were inserted 5′ of the SacI-site: Poliovirus sequence U_1_-G_9_ in pPlus9; for pPlus20 poliovirus nucleotides U_1_-A_8_, followed by C_38_-U_42_, followed by U_89_-A_95_; and for pPlus27 poliovirus nucleotides U_1_-A_8_, followed by G_35_-C_45_, followed by U_89_-A_95_. prib(+)Luc-Wt was used as parental construct for pStemA-disr and all mutants depicted in Fig. 3–4. First, the respective mutations were cloned into prib(+)Luc-Wt resulting in prib(+)Luc-N (where N is the name of the mutation), respectively, then the mutated cloverleaf sequence nucleotide U_1_-C_112_ of prib(+)Luc-N was cloned into pGC-SacI, in front of the SacI-site, resulting in pStemB-N, pStemD-N or pStemA-N, respectively. For each mutation that was engineered at the 5′ end of the poliovirus sequence, the complimentary sequence in the hammerhead ribozyme was also altered to ensure efficient cleavage. The sequences of the two cloverleaves in pdCLuc-PLuc were cloned into prib(+)Xpa resulting in pdCL-polio type 1. For stemA-mut8-virus, and stemA-mut10-virus, the sequence of the two cloverleaves and the hammerhead ribozyme in the respective replicons (p-stemA-mut8 and p-stemA-mut10), were cloned into prib(+)Xpa. CREmut-RNA has been transcribed from pCB3-CREmut, the luciferase-expressing, Coxsackie virus B3-derived replicon with a mutation within the CRE-region (A_5_ in the CRE-loop has been changed to C) as previously described [31].

Poliovirus-specific plasmid DNAs were linearized with ApaI. pCB3-CREmut was linearized with SalI. RNAs were transcribed *in vitro* in reactions containing bacteriophage T7 RNA polymerase, 5×transcription buffer [400 mM Hepes-KOH (pH 7.5), 120 mM MgCl_2_, 10 mM spermidine, 200 mM DTT] and 7.5 mM NTP-mix. After incubation at 37°C for 3 h, DNaseI (Roche) was added and reactions incubated at 37°C for 15 min. RNA was precipitated by adding 50% (in volume) of LiCl_2_-solution [7.5mM LiCl2, 50 mM EDTA (pH 8.0)] and incubation over night at −20°C. After centrifugation the pellet was washed once with 70% ethanol and then resuspended in RNA storage solution (Ambion) and stored at −80°C.

### Replication in cell-free extracts

Preparation of HeLa S10 cell extract and initiation factor has been described previously in detail [6]. Negative- and positive-strand RNA synthesis was analyzed as described before (Herold & Andino, 2000) with some minor modifications: 1 µg RNA transcripts was mixed with 25 µl HeLa S10 cell extract, 2 µl initiation factors, 5 µl 10×NTP/energy mix (Herold & Andino, 2000) and 1 µl 100mM guanidine hydrochloride in a total volume of 50 µl. After incubation at 30°C for 4h, 1 µl was removed and added to 50 µl cell culture lysis reagent (Promega) of which 10 µl was then used to measure luciferase activity to monitor translation. The rest of the original translation reaction was centrifuged and the pre-initiation complexes were resuspended in 25 µl labelling mix, containing 15 µl HeLa S10 cell extract, 2.5 µl 10×NTP/energy mix, 2.5 µl of puromycin (1mg/ml) and 30 µCi [α-^32^P]-UTP (3000Ci/mmol). After incubation at 30°C for 2 h (if not indicated otherwise), the samples were mixed with 175 µl TENSK buffer [50 mM Tris/HCl (pH7.5), 5 mM EDTA, 100 mM NaCl, 1% (v/v) SDS, 200 µg/ml proteinase K] to stop the reaction. After incubation at 37°C for 2 h, RNA was extracted with phenol/chloroform, and precipitated with ethanol. The pellet was resuspended in RNA-storage solution (Ambion) and gel-loading buffer was added prior to loading on a 0.8% agarose gel. The gel was run at 20 V constant current over night. After drying the gel, products were visualized by using autoradiography. Bands were quantified using a phosphorImager (Typhoon 9400; GE Healthcare)

### RNA transfection for luciferase timecourse

HeLa S3 cells were trypsinized, washed three times with phosphate-buffered saline, and adjusted to 5×10^6^ cells/ml. Then 800 µl aliquots were electroporated in 0.4 cm cuvettes with 20 µg of replicon RNA, using an Electro Cell Manipulator 600 (BTX Inc.) with the following settings: 300 V, 1000 µF, 24 Ω. Subsequently, 10 volumes of medium was added, the cells were divided in half, and guanidine hydrochloride (Sigma) was added to one half to a final concentration of 2 mM. 2×10^5^ cells were plated per well in 12-well plates and incubated at 37°C in a 5% CO_2_ incubator.

### Luciferase expression

Replicon-transfected cells were scraped off, washed once with phosphate-buffered saline, and then lysed in 100 µl cell culture lysis reagent (Promega). Luciferase activity in 10 µl of lysate was determined in a luminometer using the luciferase assay system (Promega).

### VPg-uridylylation-assay

HeLa S10 cell extract was programmed with replicon RNA as described in the section “Replication in cell-free extract”. Pre-initiation complexes were resuspended as decribed above but incubated for 1 h rather than 2 at 30°C. The synthesis of VPg-pUpU was then analyzed by imunoprecipitation. Briefly, 500 µl Dynabeads-ProteinA (Invitrogen) were washed twice with 0.1 M Na PO_4_ buffer [pH8.0] and then resuspended in 500 µl of the same buffer. 125 µl of anti-VPg polyclonal antibodies were added to the Dynabeads-ProteinA and incubated rotating for 1 h at room temperature. The Dynabeads were washed twice again and resuspended in 500 µl 0.1 M NaPhosphate buffer [pH8.0]. After the 1 h incubation of the replication reaction as described above, 2.5 µl of 0.5 M NaPhosphate buffer and 25 µl of the Dynabeads-ProteinA coupled with anti-VPg antibodies were added. After incubation rotating at 4°C for 1 h, the Dynabeads were washed four times with phosphate-buffered saline. The Dynabeads were resuspended in 15 µl of tricine-sample buffer (Bio-Rad). The samples were heated at 94°C for 5 min and the supernatant was run on a 20% tris-tricine gel at 75 mA for 1 h and then for 72 h at 11 mA at 4°C. After drying the gel, products were visualized by using autoradiography using a phosphorImager (Typhoon 9400; GE Healthcare).

### Growth curve

6-well plates were seeded with 10^6^ HeLa S3 cells/well and incubated over night. Cells were washed with phosphate-buffered saline and infected with a multiplicity of infection of 10 of either WT-polio type 1 or dCL-polio type 1 viruses (P0-virus) in serum-free medium. After incubation at 37°C for 30 min, cells were washed twice with phosphate-buffered saline and fresh medium supplemented with 3% newborn calf serum was added to each well. The plates were incubated at 37°C and 5% CO_2_. At indicated time points viruses were harvested by three freeze/thaw cycles followed by centrifugation. The supernatant contained the virus and was stored at −80°C. The titers of the virus were determined according to standardplaque assays [44].

### Virus production, plaque-purification and sequencing of poliovirion RNA

*In vitro* RNA transcript of viruses were transfected into HeLa S3 cells as described above in section “Cells and viruses”. Cells were incubated for 72 hours or until total cytopathic effect was reached. Viruses were harvested as described under section “growth curve.” Standard plaque-assays were performed [44]. For plaque-purification of viruses individual plaques were transferred to 6-well-plates (seeded with 10^6^ cells/well the night before) before staining of the wells. The 6-well plates were incubated for 72 hours. Total RNA was purified by tryzol extraction (Invitrogen) and isopropanol precipitation. cDNA was synthesized using the Thermoscript RT-PCR system for First-Strand cDNA Synthesis (Invitrogen). Using standard PCR techniques and specific primers for the poliovirus genome, the viral genomes were amplified and the resulting PCR products were sequenced. For sequencing of the very 5′-end of the viral genome the 5′RACE system for rapid amplification of cDNA ends (Invitrogen) was used according to the manufacturer's instructions to amplify the 5′-ends and the resulting PCR products were sequenced.

## Supporting Information

Figure S1 Structural probing of the tandem cloverleaf structures of dCL-PLuc. RNA transcripts spanning the two cloverleaf region of dCL-PLuc were 5′-labeled with [γ^32^P]ATP. The probe was digested with decreasing amounts of either RNase A, RNase T1 or RNase V1. The digested RNA was precipitated and separated on a sequencing gel. The gel was dried and autoradiographed for visualization of product using a phosphorimager. The autoradiograph of a representative mapping acrylamide gel is shown. The corresponding area of the two cloverleaves and the linker region is shown on the left side; the corresponding area within the cloverleaf structure is indicated on the right side of the autoradiograph. Major ribonuclease cleavages are indicated by the corresponding nucleotide of the cloverleaf RNA starting at the 5′-end. The very left lane (OH-Ladder) contains a hydroxyl radical 1bp produced from the same probe. The very right lane contains probe without any enzyme as a negative-control.
(3.45 MB EPS)

Figure S2 VPg-uridylylation in a cell-free replication system. RNA transcripts corresponding to tandem cloverleaf replicons containing mutations in eitherStemB, StemD, or StemA were employed to program cell-free replications systems. VPg-pU(pU) formation was monitored by incubating the extracts with [α^32^P]UTP for 1 hour. The radiolabeled RNA was immuno-precipitated using anti-VPg antibodies, separated on a Tris-Tricine SDS-Page gel and visualized by using autoradiography.
(0.44 MB EPS)
